# Tumor Characteristics and D2 Lymph Node Involvement in Gastric Cancer: A Clinicopathological Analysis (2018–2023) -

**DOI:** 10.31661/gmj.v14i.3949

**Published:** 2025-10-31

**Authors:** Vahid Zangouri, Omid Akbari Alateimouri, Hamid Zaferani Arani, AmirAli Ghahramani

**Affiliations:** ^1^ Surgical Oncology Division, General Surgery Department, Shiraz University of Medical Sciences, Shiraz, Iran; ^2^ Transplant Department, Shiraz University of Medical Sciences, Shiraz, Iran; ^3^ Surgery Department, Gilan University of Medical Sciences, Rasht, Iran; ^4^ General Surgery Department, Shiraz University of Medical Sciences, Shiraz, Iran

**Keywords:** Stomach Neoplasms, Lymph Node Excision, Survival Analysis, Lymphatic Metastasis, Neoplasm Grading, Retrospective Studies

## Abstract

**Background:**

Gastric adenocarcinoma is a leading cause of cancer-related mortality worldwide, with lymph node involvement, particularly at the level 2 lymph node dissection (D2), serving as a critical determinant of prognosis and surgical strategy. This study aimed to evaluate the association between primary tumor characteristics and D2 lymph node involvement and examine these factors’ impact on overall survival (OS) and disease-free survival (DFS) in patients undergoing curative gastrectomy.

**Materials and Methods:**

A retrospective cohort study was conducted on 233 patients with histologically confirmed gastric cancer who underwent curative-intent surgery at Namazi Hospital (Shiraz, Iran) between April 2018 and March 2023. Clinicopathological variables, including tumor size, location, grade, and histologic type, were assessed with D2 lymph node involvement. Survival outcomes were analyzed using Kaplan– Meier estimates and compared using the log-rank test. Multivariate logistic regression and Cox proportional hazards models were employed to identify independent nodal involvement and survival predictors.

**Results:**

D2 lymphadenectomy in 38.1% of patients indicated no significant associations between D2 involvement and tumor grade (P=0.443), size (P=0.215), or location (P=0.522). However, D2 lymph node metastasis was associated with a significantly descending mean of overall survival (25.43 ± 3.36 months) compared to patients without D2 involvement (43.06 ± 2.59 months; P0.001). Tumor stage and size were strong predictors of survival, with Stage 3C patients revealing a median overall survival of 13.45 months and tumors 3 cm being associated with superior outcomes (P=0.002).

**Conclusion:**

D2 lymph node involvement reflects progressive disease biology and is an assertive prognostic marker in gastric adenocarcinoma. While tumor grade, size, and location were not independently predictive of D2 metastasis, tumor stage and nodal status were strongly associated with survival. These results reinforce the use of extended lymphadenectomy in selected patients and underscore the requirement for individualized surgical planning based on total tumor profiling.

## Introduction

Gastric cancer continues to pose a serious threat to global health, being the fifth most common malignancy and the third leading cause of cancer-related mortality worldwide [1]. Based on the GLOBOCAN database, approximately 1.089 million new cases were diagnosed in 2020 alone, resulting in 769,000 deaths [2]. The burden is most noticeable in Eastern Asia, where approximately 75% of all instances occur. Iran records an annual incidence of roughly 7,300 new cases. The predominant histological type, adenocarcinoma, accounts for approximately 90% of all gastric cancer [3][4]. It follows the Lauren classification, which categorizes tumors into intestinal and diffuse types, each with distinct clinical behaviors and prognostic implications [5].

Lymph node involvement is a paramount factor in gastric cancer staging and prognosis. Surgical management is the cornerstone of curative treatment for gastric cancer, with lymphadenectomy designating a critical component of the surgical approach [6]. The extent of lymph node dissection, particularly D2 lymphadenectomy, directly affects staging accuracy and patient outcomes. Most experts agree that even localized gastric tumors should undergo at least a D1 dissection (removal of perigastric nodes). In contrast, in high-incidence regions (e.g., Japan and South Korea), a formal D2 lymphadenectomy (removal of second-tier nodes) has become standard practice. Although early Western trials did not show a clear survival advantage for D2 over D1 dissection (likely due to higher operative morbidity in the D2 arms), more recent analyses suggest that extended nodal clearance may improve long-term survival in selected patients. Thus, optimizing lymphadenectomy is a key focus in modern gastric cancer treatment [7]. The decision to perform D1 versus D2 lymphadenectomy was individualized based on a combination of patient-specific factors, tumor characteristics, and institutional practice. At our center, D1 dissection was routinely performed for early-stage tumors or when patient comorbidities, age, or intraoperative findings limited the feasibility of a more extended dissection. In contrast, D2 lymphadenectomy was preferred for patients with clinically advanced disease, good performance status, and no contraindications to more extensive surgery. Although no formal institutional protocol mandated the extent of dissection, the surgical team adhered to the principles outlined by the Japanese Gastric Cancer Association (JGCA) guidelines [8], and decisions were made in consensus among senior surgeons with subspecialty expertise. Surgeon experience and intraoperative judgment also played a role, especially in borderline cases. This selective approach reflects evolving international practices, where extended nodal clearance is increasingly recommended for staging accuracy and potential survival benefit in appropriately selected patients.

Gastric cancer has been further categorized into four molecular subtypes: Epstein-Barr virus-infected, microsatellite instability, genomically stable, and chromosomally unstable. This classification system allows for more tailored treatments by associating predicted outcomes with how patients respond to therapy [9]. Elucidating the specific relationship between primary tumor characteristics and lymph node metastasis patterns optimizes surgical strategies and improves patient outcomes. Tumor size, invasion depth, and histopathological subtype features significantly affected lymphatic spread [10]. Identifying reliable predictors of nodal spread is of evident clinical importance. If certain tumor features could accurately stratify patients by their risk of D2 involvement, surgeons could confidently tailor the extent of lymphadenectomy accordingly [11]. Normally, assured identification of low-risk tumors might allow less-extensive discarding, whereas high-risk cases (extensive or poorly differentiated tumors) would encourage extensive D2 dissection. Indeed, recent studies recommending routine D2 dissection due to the unpredictable metastasis patterns, correlating tumor traits with nodal status, could direct patient preference [12]. Such correlations could refine tumor staging and treatment planning, benefiting patients most likely to respond to surgery and adjuvant therapy and reducing the risk of complications.

Despite advances in diagnosis and treatment, gastric cancer survival rates remain dismal at about 20% five years after diagnosis, emphasizing the urgent need for improved prevention and treatment strategies. Hence, a deeper understanding of the intricate relationship between tumor characteristics and D2 lymph node involvement will inform surgical decision-making processes and improve patient care in this challenging malignancy. This study seeks to clarify these associations to develop valuable individualized treatment strategies that maximize survival while minimizing the morbidity associated with extensive surgery. The goal is to identify specific tumor features that correlate with lymph node metastasis and influence survival outcomes among patients diagnosed with gastric cancer between 2018 and 2023.

## Materials and Methods

### Study Design and Ethical Approval

This investigation employed a retrospective cohort study design to analyze the association between tumor characteristics -including location, histologic type, size, and grade - and D2 lymph node involvement in gastric cancer patients. Furthermore, the study evaluated overall survival (OS) and disease-free survival (DFS) with lymph node involvement, stratifying patients based on the presence or absence of pathologically confirmed D2 lymph node metastasis. Ethical approval for this study was granted by the Institutional Review Board of Shiraz University of Medical Sciences (Approval Code: IR.SUMS.MED.REC.1402.057). The research adhered to the ethical principles outlined in the 1964 Declaration of Helsinki and its later amendments. Before participating, all individuals provided written informed consent after receiving a detailed explanation of the study's purpose, methods, possible risks, and expected benefits. Stringent measures were enforced at every study stage to safeguard participant privacy and data security.

### Patient Selection and Study Population

We retrospectively analyzed data from patients diagnosed with histologically confirmed gastric cancer who underwent curative-intent surgical resection at Namazi Hospital (Shiraz, Iran) between April 2018 and March 2023. Patient selection was conducted through a comprehensive census approach within the specified timeframe. Eligible participants had complete pathological staging documentation including, tumor size, location, histological grade, depth of invasion, and lymph node status.

Inclusion criteria comprised: (1) histologically confirmed gastric adenocarcinoma, (2) complete medical records including preoperative imaging, operative reports, and pathological findings, and (3) signed informed consent for study participation.

Exclusion criteria were: (1) Non-adenocarcinoma histology, (2) Incomplete pathological staging data, (3) Receipt of neoadjuvant chemotherapy or radiotherapy, (4) Presence of synchronous malignancies, (5) R2 resection (macroscopically incomplete resection).

### Clinical and Pathological Assessment

Clinical and pathological variables extracted from electronic medical patient records using a standardized data collection form. These variables included living status, tumor stage, tumor location, histologic type, tumor grade, tumor size, lymph node dissection type, margin status, presence of perineural and lymphovascular invasion, peritoneal seeding, liver metastasis, type of surgery, recurrence, and administration of chemotherapy or radiotherapy.

The tumor stage was determined based on the 8th edition of the American Joint Committee on Cancer’s (AJCC) TNM classification system [13]. AJCC provides consensus criteria for staging gastric carcinoma based on tumor invasion depth, nodal involvement, and metastasis.

Tumor location was defined anatomically as proximal, distal, lesser curvature, or greater curvature, after surgical and radiological patterns of upper gastrointestinal, and tumor size ranking as <3 cm, 3-6 cm, >6 cm [14][15]. Histologic classification was based on WHO (World Health Organization) criteria, tumors categorized as well-differentiated, poorly differentiated, or signet-ring cell type [16]. Tumor grade was assigned as Grade 1 - 3 (for well-differentiated, moderately differentiated, or poorly differentiated), consistent with histopathological grading guidelines [17]. Margin status was defined as tumor-free or involved based on the pathological evaluation of the proximal and distal. The presence or absence of perineural and lymphovascular invasion was documented according to histological criteria defined in routine gastrointestinal pathology [18].

The type of lymphadenectomy (D1 or D2) and extent of gastrectomy (total or distal) were documented, following the guidelines of the JGCA, as D2 dissection includes removal of perigastric and second-tier nodes (stations 1-12) [8].

Treatment data, such as chemotherapy and radiotherapy, were recorded in patient documents. Recurrence status was assessed during follow-up visits through clinical, radiological, or endoscopic evaluation, consistent with oncologic surveillance protocols [19].

### Surgical Procedure and Pathological Examination

Board-certified surgical oncologists with expertise in upper gastrointestinal cancers performed all surgical procedures by endoscopic submucosal dissection. The operating surgeon precisely dissected lymph nodes from the surgical specimen and submitted them individually in labeled containers corresponding to their anatomical stations [20].

All resected lymph nodes were sectioned at 2-mm intervals, stained with hematoxylin and eosin, and microscopically explored for metastatic involvement. Immunohistochemical staining was performed when necessary to confirm metastasis [20]. The lymph node ratio was computed as the number of metastatic lymph nodes divided by the total number of examined lymph nodes. Surgical margins were assessed to assure the completeness of resection, and the primary tumor was evaluated for size, histological subtype, grade, and the presence of perineural and lymphovascular invasion. Pathological examination was conducted independently by expert gastrointestinal pathologists blinded to the clinical results.

### Follow-up Protocol and Outcome Assessment

Patients were systematically followed according to a standardized protocol: clinical examinations every three months for the first two years post-surgery and subsequently at six-month intervals for up to five years. Follow-up evaluations included physical examination, laboratory tests including tumor markers (CEA, CA 19-9), contrast-enhanced CT scans of the chest, abdomen, and pelvis (every six months for the first two years, then annually), and surveillance upper endoscopy at one- and three-years post-surgery.

The primary outcomes were the overall survival (OS), the period from surgery to death for any cause, and disease-free survival (DFS), the time from the surgery date to the first recorded recurrence or death, whichever came first. Clinical and radiological observations categorized recurrence patterns as distant, peritoneal, or locoregional metastases.

### Statistical Analysis

Data analysis was performed using SPSS software version 26.0 (IBM Corp., Armonk, NY). Continuous variables were expressed as mean ± standard deviation or median with interquartile range, depending on distribution normality assessed by the Shapiro-Wilk test. Categorical variables were presented as frequencies and percentages.

Associations between tumor characteristics and D2 lymph node involvement were assessed by chi-square or Fisher's exact test for categorical variables and independent t-test or Mann-Whitney U test for continuous variables. Multivariate logistic regression was employed to identify independent predictors of D2 lymph node metastasis, with results represented as odds ratios with 95% confidence intervals.

Survival analyses were performed using the Kaplan-Meier method, with differences between groups evaluated by the log-rank test. Overall survival was defined as the interval between surgery and death for any reason. In contrast, disease-free survival represented the time from surgery to disease recurrence or death, whichever occurred first. Cox proportional hazards regression was utilized to determine factors independently associated with survival outcomes after adjusting for potential confounders. A two-sided P <0.05 was regarded as statistically significant for all analyses.

### Study Limitations

A prominent limitation of this investigation was the occasional incompleteness of patient forms, which may have affected the comprehensiveness of specific variables and follow-up inspections. The retrospective nature of some data collection further highlights the need for cautious interpretation of results.

## Results

**Table:** Table1. Baseline Clinicopathological Characteristics of the Study Population (n=233)

**Variable**	**n (%)**	**Variable**	**n (%)**
**Living status**		**Proximal margin status**	
Alive	142 (60.9)	Tumor-free	346 (92.0)
Deceased	91 (39.1)	Involved	30 (8.0)
**Tumor stage**		**Tumor grade**	
1A	32 (9.0)	Grade 1	66 (22.8)
1B	21 (5.9)	Grade 2	77 (26.6)
2A	61 (17.2)	Grade 3	147 (50.7)
2B	78 (22.0)	**Histologic type**	
3A	35 (9.9)	Well-differentiated	166 (45.2)
3B	72 (20.3)	Poorly differentiated	121 (33.0)
3C	50 (14.1)	Signet-ring cell	80 (21.8)
4	5 (1.4)	**Type of surgery**	
**Lymph node dissection**		Total gastrectomy	232 (57.6)
D1	341 (61.9)	Distal gastrectomy	171 (42.4)
D2	210 (38.1)	**Peritoneal seeding**	
**Distal margin status**		Yes	19 (8.8)
Tumor-free	351 (93.1)	No	197 (91.2)
Involved	26 (6.9)	**Liver metastasis**	
**Perineural invasion**		Yes	5 (2.5)
Present	177 (47.7)	No	195 (97.5)
Absent	194 (52.3)	**Recurrence**	
**Lymphovascular invasion**		Yes	67 (35.1)
Present	196 (52.7)	No	124 (64.9)
Absent	176 (47.3)	**Chemotherapy**	
**Tumor site**		Yes	150 (59.3)
Proximal	70 (22.5)	No	103 (40.7)
Distal	124 (39.9)	**Radiotherapy**	
Lesser curvature	90 (28.9)	Yes	63 (25.6)
Greater curvature	27 (8.7)	No	183 (74.4)

Percentages are based on available data. “Tumor-free” refers to histologically negative margins. Staging follows AJCC 8th edition.

**Table:** Table2. Correlation of the D2 Lymph Node Involvement and Tumor Characteristics

**Variables**	**D2 lymph node**		**P-values**
	Yes	No	
**Tumor grade (290)**			0.443
1	32 (48.5)	34 (51.5)	
2	42 (54.5)	35 (45.5)	
3	67 (45.6)	80 (54.4)	
**Location (in CT scan) (138)**			0.522
Proximal	24 (77.4)	7 (22.6)	
Distal	52 (63.4)	30 (36.6)	
Lesser curvature of stomach	18 (72)	7 (28)	
Greater curvature of stomach	6 (66.7)	3 (33.3)	
**Tumor size (342)**			0.215
< 3 cm	51 (60)	34 (40)	
3-6 cm	85 (50.9)	82 (49.1)	
> 6 cm	45 (47.4)	50 (52.6)	

Values represent number of cases (%). P-values were calculated using the chi-square test. Total cases per variable are indicated in parentheses.

**Table:** Table3. Cox Proportional Hazards Regression for Predictors of Overall Survival (n=233)

**Variable**	**Hazard Ratio (HR)**	**95% CI**	**P-value**
**D2 lymph node involvement**	2.06	1.36 - 3.13	<0.001
**Tumor size > 6 cm**	1.57	1.01 - 2.46	0.045
**Poorly differentiated grade**	1.23	0.79 - 1.93	0.360
**Peritoneal seeding**	2.94	1.51 - 5.73	0.001
**AJCC Stage ≥ 3**	3.86	2.02 - 7.36	<0.001
**Receipt of chemotherapy**	0.72	0.47 - 1.11	0.139

Model adjusted for age, surgical type, and recurrence status; Global model P<0.001

**Table:** Table4. Multivariate Logistic Regression for Predictors of D2 Lymph Node Involvement

**Variable**	**Odds Ratio (OR) **	**95% CI**	**P-value**
**Tumor size > 6 cm **	1.92	1.01 - 3.65	0.045
**Poorly differentiated grade **	1.33	0.71 - 2.50	0.378
**Presence of lymphovascular invasion **	2.48	1.27 - 4.82	0.007
**Perineural invasion **	1.67	0.89 - 3.14	0.111
**Tumor located on lesser curvature **	1.22	0.62 - 2.41	0.560
**AJCC Stage ≥ 3 **	3.14	1.42 - 6.91	0.004

Model summary: Nagelkerke R²=0.29; Hosmer–Lemeshow P=0.52

**Table:** Table5. Survival of the Participants based on the Tumour Staging

**Stage**	**Total N**	**N of events**	**Survival**	**Median time (month)**	**P-value**
**Stage 1A**	18	0	100.0%	69	
**Stage 1B**	12	3	75.0%	60	
**Stage 2A**	31	9	71.0%	57	
**Stage 2B**	46	17	63.0%	43.9	< 0.001
**Stage 3A**	16	4	75.0%	38.11	
**Stage 3B**	36	19	47.2%	22.98	
**Stage 3C**	25	19	24.0%	13.45	

Survival rates and median survival times are based on Kaplan–Meier analysis. “N of events” refers to the number of deaths. P-value calculated using the log-rank test.

**Figure-1:**
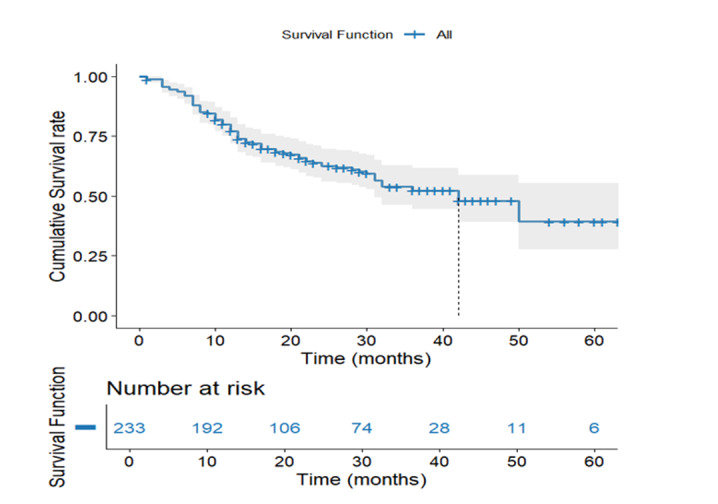
Figure 1. Overall Survival Kaplan-Meier Curve. Overall survival curve depicting cumulative survival of all participants with gastric adenocarcinoma

**Figure-2:**
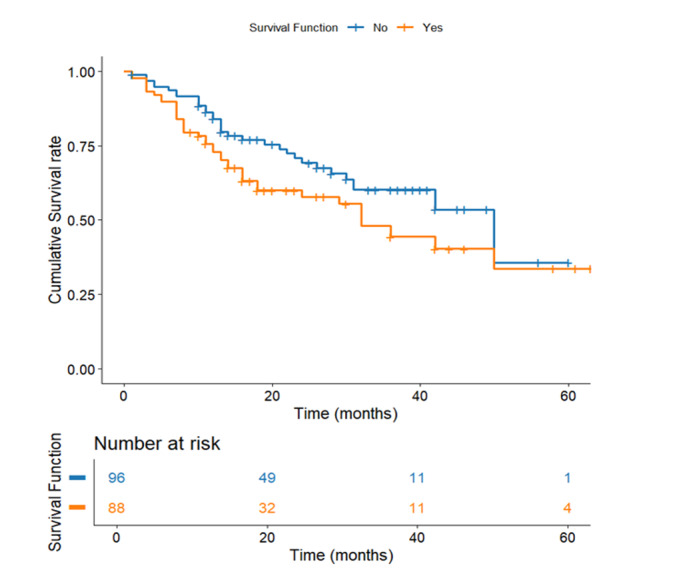
Figure 2. Survival Based on D2 Lymph Node Involvement. Kaplan-Meier survival curve comparing patient survival according to D2 lymph node involvement

**Figure-3:**
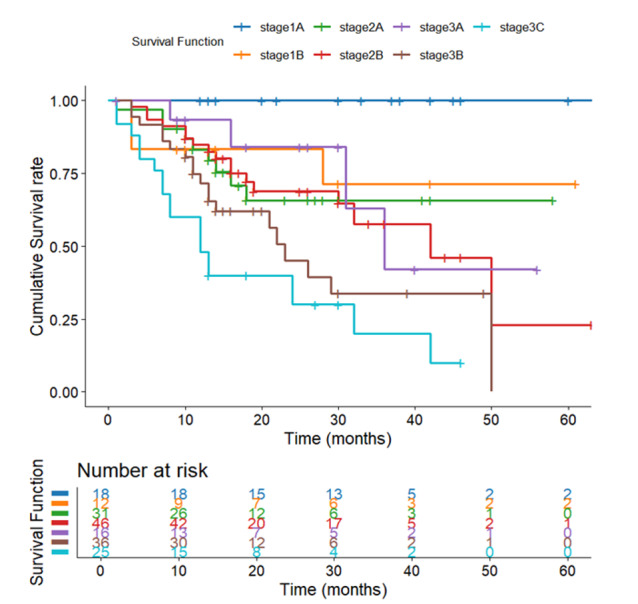
Figure 3. Survival Stratified by Tumor Staging. Kaplan-Meier survival curve illustrating survival differences across tumor stages

**Figure-4:**
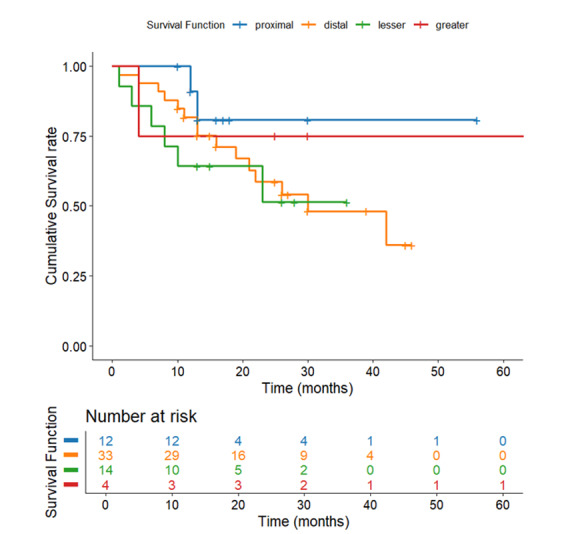
Figure 4. Survival Based on Tumor Location. Kaplan-Meier survival curve showing survival variations by tumor location on CT scan

**Figure-5:**
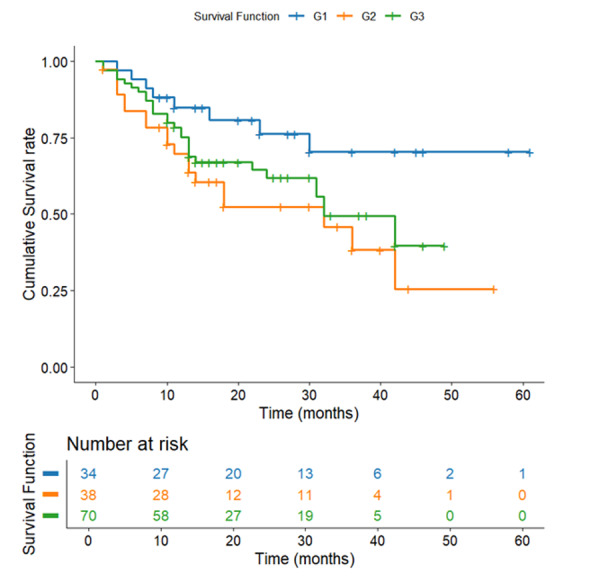
Figure 5. Survival Stratified by Tumor Grade. Kaplan-Meier survival curve depicting survival differences across tumor grades.

**Figure-6:**
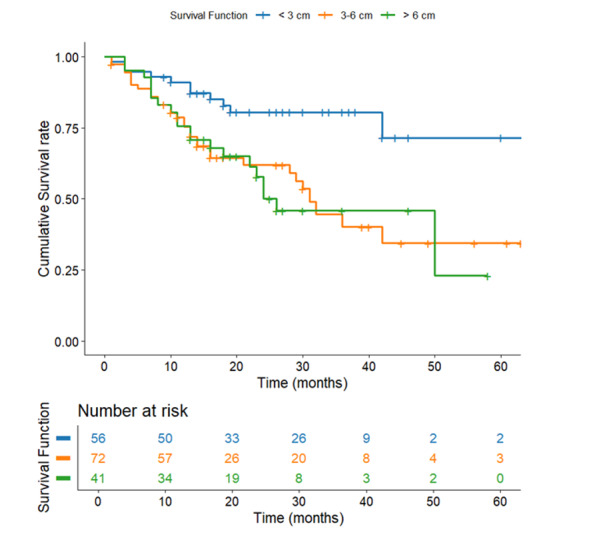
Figure 6. Survival Based on Tumor Size. Kaplan-Meier survival curve depicting survival differences across tumor grades (n=169)

**Figure-7:**
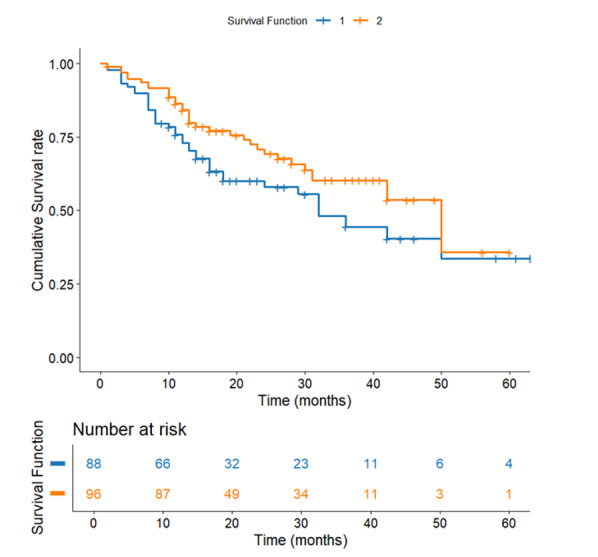
Figure 7. Survival Comparison of Lymph Node Dissection Types. Kaplan-Meier survival curve comparing survival outcomes between D1 and D2 lymph node dissection (n=184)

### Clinicopathologic Patient Characteristics

A total of 233 patients with histologically confirmed gastric cancer were included in the study. The baseline characteristics of the study population were gathered (Table-1). At the time of analysis, 60.9% of the patients were alive, while 39.1% were deceased. Most patients were diagnosed at an advanced stage, with the most significant proportion classified as Stage 2B (22%), followed by Stage 3B (20.3%) and Stage 3C (14.1%), and early-stage disease (Stages 1A and 1B) accounted for only 14.9% of the cohort.

D2 lymph node dissection was accomplished in 38.1% of cases, while the remaining 61.9% underwent D1 dissection. Histopathological evaluation demonstrated that 93.1% of patients had tumor-free distal margins, and 92% had tumor-free proximal margins. Perineural invasion was observed in 47.7% of patients, and lymphovascular invasion was identified in 52.7% of cases.

The distal part of the stomach (39.9%) was the most regular tumor location, followed by the lesser curvature (28.9%) and the proximal region (22.5%). Tumor grading showed that poorly differentiated tumors were predominant (50.7%), followed by moderately differentiated (26.6%) and well-differentiated tumors (22.8%). Regarding histological subtypes, 45.2% of tumors were well-differentiated adenocarcinomas, 33% were poorly differentiated, and 21.8% were signet-ring cell carcinomas.By surgical interventions, 57.6% of patients underwent total gastrectomy, whereas 42.4% received distal gastrectomy. Imaging analyses (CT scans) verified that 55.8% of tumors were located distally, with proximal tumors accounting for 21.1% of cases. In 8.8% of patients, peritoneal seeding was present, but liver metastasis was infrequent (2.5%). Recurrence occurred in 35.1% of cases. Systemic treatment was distributed variably: 59.3% of patients received chemotherapy, whereas 25.6% underwent radiotherapy. The relatively low rate of radiotherapy usage implies the heterogeneity of treatment protocols within this population.

### Overall Survival

The relationship between D2 lymph node dissection and tumor characteristics was assessed (Table-2). There were no statistically significant associations between D2 dissection and tumor grade (P=0.443), tumor location as seen on CT scan (P=0.522), or tumor size (P=0.215).

Particularly, D2 dissection rates were similar across tumor grades (Grade 1: 48.5%, Grade 2: 54.5%, Grade 3: 45.6%). In addition, tumor location did not immensely impact the chance of receiving D2 dissection, with relative distributions across proximal, distal, and curvature-based tumors. Likewise, tumor size did not appear to affect the dissection type, with slightly higher D2 rates observed in tumors <3 Cm, but not statistically significant. These findings indicate that D2 lymphadenectomy was not determined by tumor grade, anatomical location, or size in this cohort.

In every case of gastric adenocarcinoma, the overall survival curve indicates a gradual decline in survival probability over time (Figure-1). Accordingly, the overall survival rate was 60.9%, with a mean survival of 40.71 months (SE=2.30), indicating a heterogeneous gastric cancer prognosis. According to this curve, mortality rates are invariant during follow-up due to their steady progression, without harsh declines.

This trend is consistent with the study population's characteristics, with most patients (77.1%) diagnosed at advanced stages (Stage 2B and above), establishing this survival pattern. Initially, the reduction was sharp, presumably describing high-risk subgroups, while the later plateau implies varying progression rates.

Multivariate Cox proportional hazards regression analysis was conducted to identify independent predictors of overall survival (Table-3).

D2 lymph node involvement was significantly associated with worse survival (HR=2.06, 95% CI: 1.36-3.13, P<0.001), indicating that patients with second-tier nodal metastasis had more than double the risk of mortality compared to those without. Advanced tumor stage (Stage ≥ III) was the strongest predictor of poor outcome (HR=3.86, 95% CI: 2.02-7.36, P<0.001), followed by the presence of peritoneal seeding (HR=2.94, P=0.001). Tumor size greater than 6 cm was also independently associated with decreased survival (HR=1.57, P=0.045). In contrast, histologic grade and chemotherapy administration were not significantly correlated with overall survival in the adjusted model.

These findings underscore the prognostic impact of disease burden, especially nodal and peritoneal dissemination, and support the need for aggressive management in patients with high-risk features.

Survival Based on D2 Lymph Node Involvement

In gastric adenocarcinoma, lymph node status is critical in determining survival disparities between patients with and without involvement of D2 lymph nodes. The analysis reveals a significant 18-month survival difference, with patients without D2 involvement having significantly longer mean survival (43.06 months, SE=2.59) versus those with D2 involvement (25.43 months, SE=3.36; P<0.001). The early and sustained divergence of curves shows D2 metastases as a critical prognostic factor, likely due to greater disease burden and aggressive biology (Figure-2).

Multivariate logistic regression analysis identified several independent predictors of D2 lymph node involvement (Table-4). The presence of lymphovascular invasion significantly increased the odds of D2 metastasis (OR=2.48, 95% CI: 1.27-4.82, P=0.007), as did advanced tumor stage (Stage ≥ III) (OR=3.14, 95% CI: 1.42-6.91, P=0.004). Tumor size >6 cm was also a significant predictor (OR=1.92, P=0.045). In contrast, tumor grade, perineural invasion, and tumor location were not independently associated with D2 involvement. These findings suggest that deeper invasion and vascular spread, rather than histologic grade or site, are more indicative of advanced nodal metastasis.

### Survival Stratified by Tumor Staging

Kaplan-Meier analysis revealed that overall survival was significantly associated with tumor stage (P<0.001, Figure-3). Patients with early-stage gastric cancer (Stages 1A and 1B) exhibited the most favorable outcomes, with Stage 1A leading to 100% survival and a median survival time of 69 months. In contrast, survival decreased progressively with the advancing stage, and patients in Stage 3C exhibited the poorest prognosis, with only 24% survival and a median survival of 13.45 months (Table-5).

### Survival Based on Tumor Location

While not statistically significant (P=0.077), distinct survival patterns appeared by location: greatest curvature tumors displayed the best outcomes (52.75 months, SE=14.07), followed by proximal (49.98 months, SE=3.96), distal (30.28 months, SE=2.75), and lesser curvature locations (20.92 months, SE=3.45, Figure-4). This 32-month range points to clinically meaningful variations that may reflect distinctions in surgical resectability, local microenvironment, or molecular profiles. The lack of statistical significance is likely a consequence of sample size limitations, warranting further investigation into location-specific biological behaviors.

### Survival Stratified by Tumor Grade

The tumor grade survival curve describes the relationship between cellular differentiation and patient outcomes (Figure-5). Well-differentiated (Grade 1) tumors demonstrated the best outcomes (46.58 months, SE=4.10), followed by poorly differentiated (Grade 3: 30.98 months, SE=2.51) and moderately differentiated (Grade 2: 28.45 months, SE=3.79). The 18-month advantage for Grade 1 tumors suggests better treatment response and less aggressive biology, though the lack of significance highlights that grade alone is an incomplete prognostic marker. Despite these apparent differences, the lack of statistical significance (P=0.07) suggests that tumor grade alone is an insufficient predictor of survival. This nuanced finding emphasizes the multifactorial nature of gastric cancer progression, where cellular differentiation interacts with numerous other clinical and molecular factors.

### Survival Based on Tumor Size

Tumor size revealed a potent inverse relationship with survival (P=0.002, Figure-6). Tumors with <3 Cm size presented superior outcomes (59.12 months, SE=3.43) compared to intermediate (3-6 Cm: 36.38 months, SE=3.06) and large tumors (>6 Cm: 32.72 months, SE=3.64). The 26-month survival interval between the smallest and largest tumors highlights the clinical implication of early detection, as smaller size presumably reflects earlier stage, diminished metastatic potential, and greater resectability.

### Survival Comparison of Lymph Node Dissection Types

D2 lymphadenectomy was associated with a statistically significant survival advantage (40.55 months, SE=2.60) compared to D1 dissection (36.85 months, SE=3.09; P=0.037, Figure-7). While the absolute 4-month difference appears modest, more extensive lymph node removal may improve outcomes through better staging accuracy and elimination of micrometastases.

## Discussion

This study scrutinized the relationship between tumor characteristics and D2 lymph node involvement in gastric adenocarcinoma, focusing on survival consequences. The findings of D2 lymph node dissection in 38.1% of cases indicated no significant association with tumor grade, location, or size. However, survival was significantly influenced by tumor stage and size, so D2 involvement is a potent negative prognostic factor for overall survival.

Although tumor size and grade are traditionally linked to lymphatic spread, they did not independently predict D2 lymph node involvement in this study. This may reflect the biological heterogeneity of gastric cancer or inconsistencies in surgical decision-making. The findings suggest that tumor morphology alone is insufficient for predicting nodal spread, and more comprehensive models incorporating molecular, imaging, and intraoperative data are needed to guide surgical planning accurately.

The observed mean overall survival of 40.71 months and a survival rate of 60.9% are relatively favourable compared to global data. Globally, the 5-year survival rates for gastric cancer vary from 20% to 40% [21]. This range varies based on the stage of diagnosis and regional differences in healthcare infrastructure and surgical expertise. In contrast, numerous centers in East Asia report relatively better outcomes (38.5%), often attributed to earlier detection and routine D2 lymphadenectomy [22]. Even new trends in gastric cancer survival show improvements, with 5-year rates increasing from 38.3% to 42.9% in 2017-2021, mainly due to advancements in treatment strategies and early detection methods [23]. However, survival rates remain significantly lower for advanced stages of gastric adenocarcinoma, with median overall survival times ranging from 11 to 17 months [24]. In contrast, some studies report higher survival rates for specific populations. For instance, a center reported a 5-year relative survival rate of 71.4% for gastric cancer patients from 2018 to 2022 [21]. This suggests that outcomes vary significantly based on geographical location, treatment protocols, and patient demographics. Our study's favourable survival rate reflects structured follow-up, a consistent surgical protocol, and proper case selection for curative-intent resection.

Notably, the mean survival time was significantly longer for patients without D2 metastasis (43.06 months) than for those with D2 metastasis (25.43 months) (P<0.001). This result is consistent with Lu et al. (2021), who observed decreased disease-free survival in patients with central nodal metastasis even after D2 clearance, implying that aggressive tumor biology is linked to D2 positivity [11]. Similarly, Xu et al. (2022) declared that ERBB2-positive gastric cancer with lymphovascular and neural invasion is more likely to metastasize to second-tier nodes and exhibit worse outcomes [25]. These findings reinforce the role of D2 status as a biological, not just anatomical, indicator of prognosis.

Although tumor location did not reach statistical significance in predicting survival (P=0.077), a clinical trend was evident: tumors in the proximal and greater curvature areas were associated with more prolonged survival, possibly due to more effective surgical exposure and lymphatic clearance. These observations are corroborated by Wang et al. (2021), who found that tumors in the lesser curvature had higher metastatic risk and recurrence due to anatomical lymphatic drainage complexity [26]. A study found that non-cardia tumors have better survival outcomes, emphasizing the importance of tumor location in treatment planning and prognosis assessment for gastric cancer patients [27].

Tumor stage, unsurprisingly, emerged as the strongest predictor of survival. Patients in Stage 1A had a 100% survival rate, while those in Stage 3C had a survival of just 24%, with a median survival of 13.45 months. This pattern is consistent with studies by Jong et al. (2022) and Huang et al. (2021), which confirmed the prognostic weight of staging, particularly regarding lymph node burden and systemic dissemination [28][29]. Another study reported that Stage I gastric cancer patients had a two-year survival rate of 95.8%, significantly higher than those with advanced stages [30]. Hence, the five-year relative survival rates for Stage I-III patients have been reported as high as 89.7% in recent studies [21]. Meanwhile, Stage III patients generally have lower survival rates, ranging from 18% to 50% depending on the dataset [31].

Fascinatingly, tumor grade did not significantly affect survival in this cohort (P=0.07), though patients with Grade 1 tumors had an 18-month survival advantage. While histologic grade is a known prognostic indicator, its independent predictive value may diminish due to other dominant factors such as lymph node involvement or invasion depth. This notion is supported by the findings of Brisinda et al. (2023), who regarded that histological differentiation alone was insufficient to predict recurrence unless paired with advanced T-stage or vascular invasion [32].

On the contrary, tumor size demonstrated a robust inverse association with survival (P=0.002). Patients with tumors smaller than 3 Cm lived nearly 27 months longer than those with tumors larger than 6 Cm. This conclusion highlights the importance of early detection. It aligns with the study of Cai et al. (2022), who developed a risk model in early gastric adenocarcinoma, indicating that tumor size was an independent predictor of lymph node metastasis. Larger tumors reflect longer subclinical evolution and increased nodal and systemic spread possibility [33].

Despite the absence of a direct correlation between tumor characteristics and the decision to perform D2 dissection, patients who underwent D2 dissection had a statistically more prolonged mean survival (40.55 vs. 36.85 months, P=0.037). This reinforces findings from Guo et al. (2024), who revealed that even in obese patients undergoing laparoscopic D2+ dissection, long-term survival was improved without raised perioperative morbidity. While D2 may not be selectively indicated based on tumor size or location alone, its survival advantage presents a more typical application that may be warranted, particularly in operable, node-positive patients [34]. However, some studies show that D2 over D1 has a survival advantage, as demonstrated in the Dutch trial's 15-year results, reducing locoregional recurrence and gastric adenocarcinoma-related deaths [35].

This study has several limitations that should be acknowledged. First, its retrospective design may introduce selection and information biases, as data collection relies on existing medical records, which may be incomplete or inconsistently documented. Second, the extent of lymphadenectomy (D1 vs. D2) was not standardized across all cases and was influenced by individual surgeon judgment, potentially affecting the comparability of outcomes. Third, molecular and genetic tumor characteristics—such as HER2 status or microsatellite instability—were not evaluated, which could have further refined prognostic stratification. Lastly, although the sample size was sufficient for primary analyses, subgroup comparisons may have been underpowered to detect smaller effect sizes.

Future prospective, multi-center studies with standardized surgical protocols and molecular profiling are warranted to validate and expand upon these findings.

Altogether, this study adds to the growing body of evidence suggesting that tumor characteristics—particularly stage, size, and lymph node status—should guide surgical and therapeutic decision-making. While traditional clinicopathologic variables such as grade and location have predictive value, they must be interpreted within a broader oncologic context. Prospective investigation should strive to integrate molecular classification systems (e.g., TCGA subtypes, MSI status) with surgical data to construct more refined, personalized treatment algorithms. The emerging use of biomarkers such as circulating microRNAs and radiomic signatures may also enhance the preoperative prognosis of nodal spread and support tailored lymphadenectomy strategies.

## Conclusion

This study highlights the prognostic significance of tumor stage, size, and D2 lymph node involvement in patients with gastric adenocarcinoma undergoing curative surgery. While no significant association was observed between tumor grade, size, or location and the likelihood of D2 dissection, extended lymphadenectomy was associated with improved survival outcomes. These findings support the continued use of D2 dissection in appropriate surgical candidates and underscore the importance of individualized treatment planning based on comprehensive pathological evaluation.

### Suggestions for Future Prospects

Future gastric cancer studies require the incorporation of advanced diagnostic approaches and personalized treatment strategies. These include precise lymph node dissection techniques, molecular markers, genetic profiling, and tailored treatment procedures. Routine screenings and increased awareness can improve prognosis and patient surveillance. This multifaceted strategy could significantly impact the future of gastric cancer treatment and research.

## Conflict of Interest

There was no conflict of interest.
